# Supplementary material for: Extraction, Structural Analysis, and Biofunctional Properties of Exopolysaccharide from Lactiplantibacillus pentosus B8 Isolated from Sichuan Pickle
Source: Foods. 2022 Aug 4;11(15):2327. doi: 10.3390/foods11152327 (PMC9367902; doi:10.3390/foods11152327)
Supplement: Supplementary file 1 [file foods-11-02327-s001.zip › foods-1831797-supplementary.pdf]

Foods

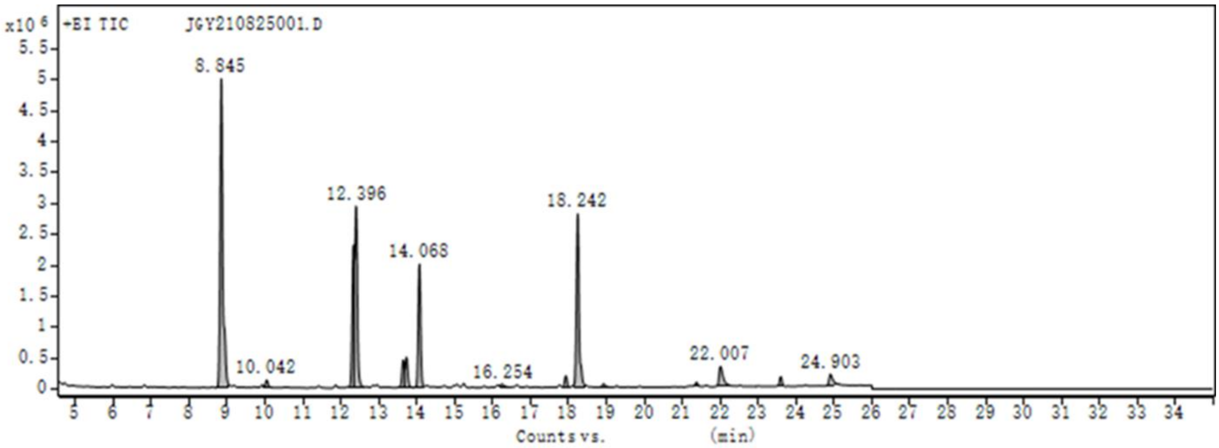

Figure S1 Methylation analysis results of LPB8-1

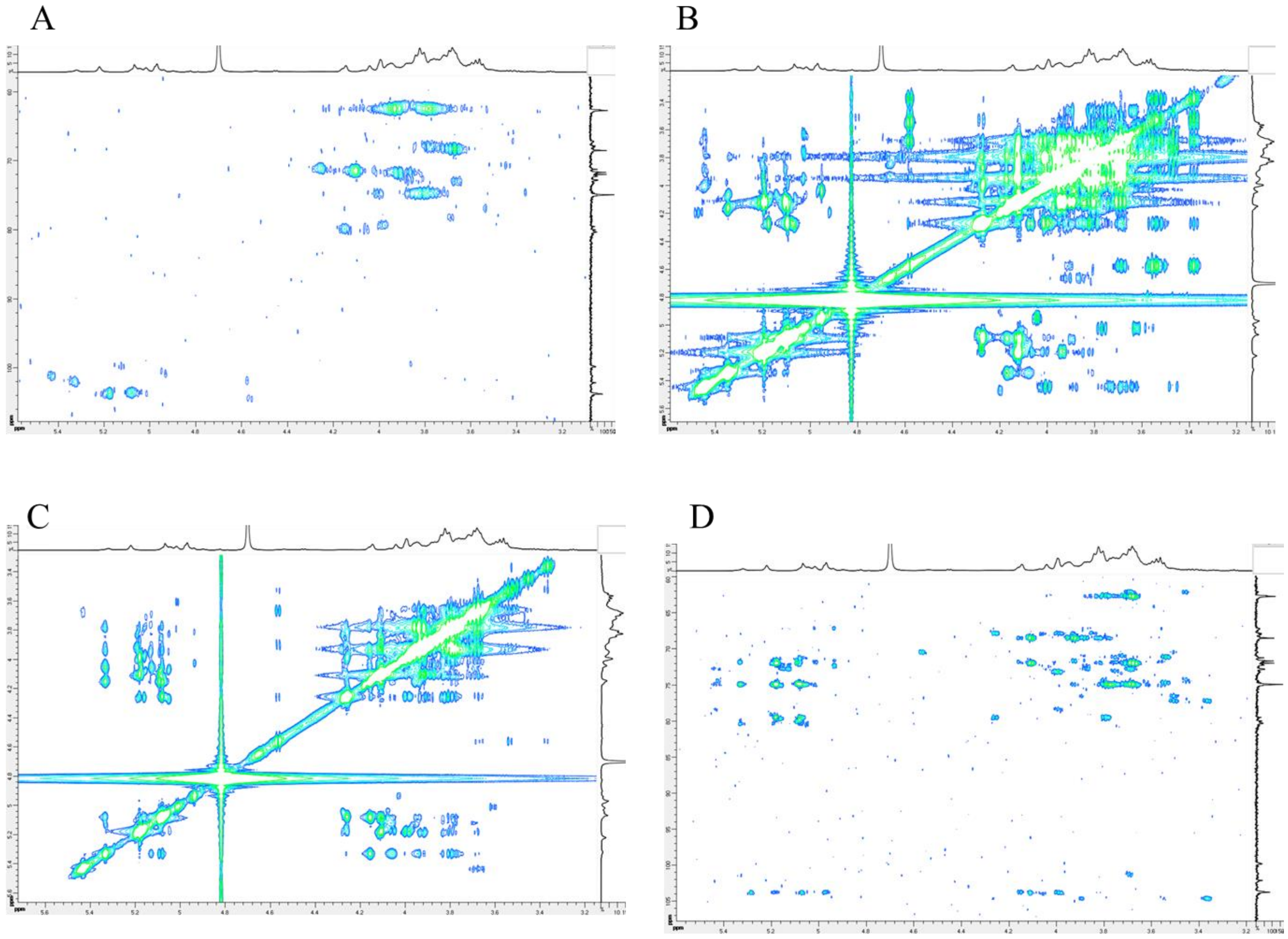

Figure S2 HSQC spectrum (A), TOCSY spectrum (B), NOESY spectrum (C), HMBC spectrum (D) of LPB8-1
